# Cultural Adaptation of the Mothers and Babies Intervention for Use in Tribal Communities

**DOI:** 10.3389/fpsyt.2022.807432

**Published:** 2022-02-17

**Authors:** Erin A. Ward, Ethleen Iron Cloud-Two Dogs, Emma E. Gier, Linda Littlefield, S. Darius Tandon

**Affiliations:** ^1^Center for Community Health, Institute for Public Health and Medicine, Feinberg School of Medicine, Northwestern University, Chicago, IL, United States; ^2^Oglala Lakota Cultural Consultant, Porcupine, SD, United States; ^3^Great Plains Healthy Start, Great Plains Tribal Leaders' Health Board, Rapid City, SD, United States

**Keywords:** pregnancy, postpartum, mental health, depression, indigenous communities, mental health intervention, cultural adaptation, Lakota

## Abstract

**Objective:**

While one in five women may experience mood and anxiety disorders during pregnancy and postpartum, Indigenous identity increases that risk by 62%, especially among younger Indigenous women. The need for evidence-based perinatal mental health interventions that provide culturally relevant well-being perspectives and practices is critical to improving maternal, child, and community outcomes for Indigenous peoples, and reducing health inequities.

**Methods:**

Through a collaboration between community maternal and child health professionals, intervention researchers, and a cultural consultant, our workgroup developed cultural adaptations to Mothers and Babies, an evidence-based perinatal depression prevention intervention. Applying a cultural interface model, the workgroup identified existing intervention content for surface adaptations, as well as deep, conceptual adaptations to incorporate traditional teachings into this evidence-based intervention.

**Results:**

This collaboration developed a culturally adapted facilitator manual for intervention providers, including guidance for implementation and further adaptation to represent local tribal culture, and a culturally adapted participant workbook for Indigenous perinatal women that reflects cultural teachings and traditional practices to promote well-being for mother and baby.

**Implications:**

Committing to a culturally respectful process to adapt Mothers and Babies is likely to increase the reach of the intervention into Indigenous communities, reengage communities with cultural practice, improve health outcomes among parents, children, and the next generation's elders, and reduce disparities among Indigenous groups. Replication of this community-engaged process can further the science and understanding of cultural adaptations to evidence-based interventions, while also further reducing health inequities. Future steps include evaluating implementation of the culturally adapted intervention among tribal home visiting organizations.

## Introduction

A collaborative group of stakeholders including a Lakota elder cultural consultant, maternal and child health professionals in Tribal communities, and perinatal mental health intervention researchers, developed a cultural adaptation of an evidence-based perinatal depression prevention intervention for use in Tribal communities. This adaptation aims to support intergenerational mental health by building resilience through cultural restoration, to better address health disparities experienced by American Indian families and communities.

## Background

Across the United States (US), 573 federally recognized Tribes represent diverse American Indian communities in urban settings and on designated reservations that are typically rural (Bureau of Indian Affairs) (1). Indigenous peoples in the US carry historical traumas and face ongoing current-day stressors. The collective history of forced displacement, attempted genocide, colonization, and dismantling of traditional family structures and cultural foundation, or “cultural assault,” has left multi-dimensional and generational lasting effects (1–3). Ongoing systemic oppression, high rates of intimate partner violence, poverty, lack of resources, and substance use create an environment ripe for increased and disproportionate risk of mental illness (4). A systematic review demonstrated that Indigenous people are at increased risk for depression, anxiety, and substance use disorders during the perinatal period, in comparison to other racial and ethnic groups (5). Moreover, intergenerational transmission of toxic stress resulting from historical trauma can perpetuate trauma reactions and further disrupt healthy bonding and parenting (2).

The effects from historical trauma contribute to contemporary traumatic experiences and exposures (6, 7). Whitbeck et al. (8) identified that anger and depression among American Indian parents was linked to experiences of multigenerational loss. This historical loss is compounded by contemporary loss that American Indians suffer (9), including having the third highest infant mortality rate in the US of any racial group (10). These risk factors are further exacerbated by under-resourced health care in American Indian communities, wherein the per person patient care allocation *via* the Indian Health Service (IHS) is approximately one-third of per person spending, nationally, among all other communities (1, 11). Moreover, while numerous validation studies have been conducted in other cultures, further work remains in developing efficacious culturally relevant perinatal depression screening tools and methods for American Indian people (4, 5, 12, 13).

Numerous barriers to accessing care include: stigma and fear of being perceived as incapable, concerns about privacy, mistrust of service providers, and lack of transportation, financial resources, and social support (1, 14, 15). Mental health care providers of Indigenous identities are underrepresented in the field, disallowing cultural congruency between provider and patient which is of known benefit (1, 16). Untreated perinatal depression can lead to adverse outcomes for parent and child. Parents who are depressed are more likely to engage in high-risk behaviors like substance use, are less likely to attend prenatal or well child visits and are more likely to suffer from future depressive episodes (17). Children of parents with depression may experience a range of negative outcomes including developmental delays, cognitive impairments, and attachment insecurity, along with increased risk for developing mental health issues (4, 18, 19).

A study of 30 American Indian teen mothers (20) found that all the women reported childhoods filled with stress which led to increased chaos in their lives. In other studies, maternal stress and risk factors including adverse experiences has shown a deleterious impact on children's social-emotional well-being (21–23). Additionally, American Indian women suffer risk factors including diabetes, tobacco use, low socioeconomic status, childhood physical abuse, rape, domestic violence, and low maternal age which contribute to preterm birth, with potential damaging consequences for child development (24). Disparities persist in the areas of health, economic, social, and education for American Indians and the intergenerational impact of historical trauma and systemic oppression as a contributing factor is an ongoing research agenda (25). The forced removal of American Indian children into boarding schools systematically stripped parents' rights to parent, a message that their families are not appropriate to raise children, with far-reaching effects on subsequent generations (2). Furthermore, American Indians have a disproportionately rate of abused children-−14.8%—while accounting for 2% of the US population (26). The Child Welfare League of America (27) reported that 46% of the children in out-of-home care in South Dakota were American Indian children.

Despite the massive historical traumatic events and current challenges experienced by American Indians, the dynamic of resilience cannot be ignored. Elders in one study identified individual, family, and community constructs that promote resilience as connectedness, culture, and spirituality (28); furthermore, as Tolliver-Lynn et al. (29) relate, “many protective characteristics that promote resilience are deeply embedded in traditional AI/AN culture” (29). Elders in another study asserted that healing from said historical traumas involves reclamation of one's culture (9, 30). In this paper, we will describe the process of culturally adapting an evidence-based perinatal depression prevention intervention, to begin to address some of these inequities through the reclamation of cultural and spiritual wellness practices within the context of parenting.

### Opportunity for Community Impact

The Great Plains Tribal Leaders' Health Board—who serve 18 Great Plains Tribal communities—received funding to support the maternal and child health of families living on or near the Rosebud Indian Reservation, South Dakota (Project LAUNCH: Linking Actions for Unmet Needs in Children's Health). A core strategy included professional development for maternal and child health outreach workers. Mothers and Babies (MB), an evidence-based perinatal depression prevention intervention frequently used in home visiting, was selected for provider training and implementation in the Great Plains Tribal communities.

### About Mothers and Babies

MB is an evidence-based perinatal depression prevention intervention, based on foundations of cognitive-behavioral therapy (CBT) and attachment theory, that provides a toolkit of cognitive, behavioral, social, and mindfulness approaches to effectively respond to stress while pregnant, preparing for, and parenting a baby or young child (31, 32). MB uses a psychoeducational approach to increase engagement in pleasant activities, promote helpful thoughts and reduce unhelpful thoughts, and strengthen social support and communication with one's social network and community. MB can be implemented as a group intervention (33–37) or individually (38, 39). Each core module (Pleasant Activities, Thoughts, and Contact with Others) is introduced by a character vignette with two pregnant/postpartum people, illustrating the connection with their mood (see Table 1). MB can be implemented by providers from various educational and professional backgrounds, in settings where pregnant people and new parents access services and has been recommended by the US Preventive Services Task Force (40, 41). MB is being scaled across the US in home visiting and other maternal and child health settings, using a four-stage process including pre-training conversations, training, implementation consultation, followed by implementation evaluation and maintenance.

**Table 1:** Mothers and babies intervention content.

**MB module**	**MB session**	**Participant worksheet**
Introductory	Session 1: Introduction to mothers and babies	1.1 *Stressors that can affect the mother–baby relationship*
		1.2 *How the Mothers and Babies course can help you*
		1.3 *What is mindfulness and breath awareness*
		1.4 *Your mood and your personal reality*
		1.5 *Quick mood scale*
Pleasant activities	Session 2: Pleasant activities and mood	2.1 *Violet and Mary's days*
		2.2 *Pleasant activities list*
		2.3 *Mindfulness practice—Body scan*
		2.4 *What do you like to do?*
	Session 3: Pleasant activities with baby	3.1 *Overcoming obstacles*
		3.2 *Make a personal commitment*
		3.3 *Personal commitment calendar*
		3.4 *How do babies learn?*
		3.5 *From birth to 1: Some things babies like to do*
		3.6 *Mindfulness practice—Walking meditation*
		3.7 *Quick mood scale and pleasant activities*
Thoughts	Session 4: Thoughts and how they affect mood	4.1 *What are thoughts?*
		4.2 *Violet and Mary's days*
		4.3 *Mindfulness practice*—*Leaves on a Stream*
		4.4 *Helpful thoughts and unhelpful thoughts*
	Session 5: Identifying and modifying unhelpful thoughts	5.1 *Unhelpful thought patterns and challenging them*
		5.2 *Ways to change unhelpful thoughts*
		5.3 *Stopping unhelpful thoughts*
	Session 6: Relationship between mood, thoughts, and the future	6.1 *Thoughts about being a mother*
		6.2 *Thinking about your baby's future*
		6.3 *Thinking about your future*
		6.4 *Quick Mood Scale and Thoughts*
Contact with others	Session 7: Contact with other people and mood	7.1 *Relationship between mood and contact with others*
		7.2 *Violet and Mary's days*
		7.3 *Mindfulness practice—Web of life*
		7.4 *Quick mood scale and contact with people*
	Session 8: Social support for parent and child	8.1 *The people in my life*
		8.2 *People in my life and the ways they support me*
		8.3 *Role changes and disagreements*
		8.4 *People who provide support for me and my baby*
	Session 9: Communication style and mood	9.1 *Communication styles and your mood*
		9.2 *Getting your needs met*

### Impetus for Cultural Adaptation

The inception of this cultural adaptation began during a live-virtual MB intervention training. Training participants included Great Plains maternal and child health leadership and supervisors, infant mental health consultant, and direct service staff. During the training, several examples within the MB manuals were identified as incongruous with typical resources and experiences among reservation communities; participants discussed benefits of incorporating traditional teachings and culturally appropriate images and examples into the MB intervention to support Native American families. An interdisciplinary team was later engaged to conduct the process of cultural adaptations to the MB intervention manuals.

## Methods

### Science of Adaptation

Evidence Based Interventions (EBI) are often designed and scaled to reduce health disparities, but tend to be tested within one community, precipitating the need for improved contextual, and cultural fit as they are replicated and scaled (42). MB is an example of an EBI that has demonstrated effectiveness in reducing depressive symptoms and major depressive episodes among perinatal people and has been tested in diverse racial and ethnic populations. However, its reach within Indigenous communities has been limited, due in part to the need for culturally appropriate adaptations to ensure fit, acceptability, adoption, and positive impact.

Cultural adaptation is “the systematic modification of an evidence-based treatment (EBT) or intervention protocol to consider language, culture, and context in such a way that it is compatible with the client's cultural patterns, meanings, and values” (43). EBIs serve diverse populations in ever-changing contexts, with adaptation allowing service providers greater ownership over the content and bringing families' culture and expertise to the forefront. Adapting an intervention with community members promotes likelihood that its implementation will be sustained (44).

There are key elements of an EBI that should be maintained, including the pedagogy of the intervention (45, 46). MB's core components are CBT and attachment theory: regardless of adaptation, participants should receive and emerge with the same skills and knowledge (47). Participant responsiveness is another a key component of fidelity, making adaptations crucial to maintaining participant engagement (47). Making “fidelity-consistent” adaptations while maintaining these core elements can ultimately impact the reach and effectiveness of the intervention across settings, changing contexts, and over time (48).

There are various types of adaptations that fall into “surface level” or “deep” adaptations (49). Surface level adaptations attempt to match intervention materials to the observable characteristics of a population, like ensuring the people in images represent those being served and incorporating elements of music, language, food, and clothing into materials. Deep adaptations incorporate cultural, social, historical, environmental, psychological, and spiritual factors that can influence the health of the population (50, 51). Our work included both surface-level and deep adaptations to MB.

### Process of Development and Workgroup Structure

The cultural adaptation process was conducted by a collaborative group of key stakeholders representing maternal and child health service providers, Lakota cultural expertise, and MB intervention researchers. Workgroup participants from the maternal and child health team included a program director, manager, supervisor, and staff member, all of whom had attended MB training, and some of whom were implementing MB or supervising implementation. An elder of the Oglala Sioux Tribe with expertise providing cultural consultation and training in areas of mental health and youth and family well-being, guided the process to incorporate traditional teachings into the MB intervention and manuals. Several members of the MB team with expertise in MB intervention development, implementation, and effectiveness research participated in the adaptation process.

**Figure 1:**
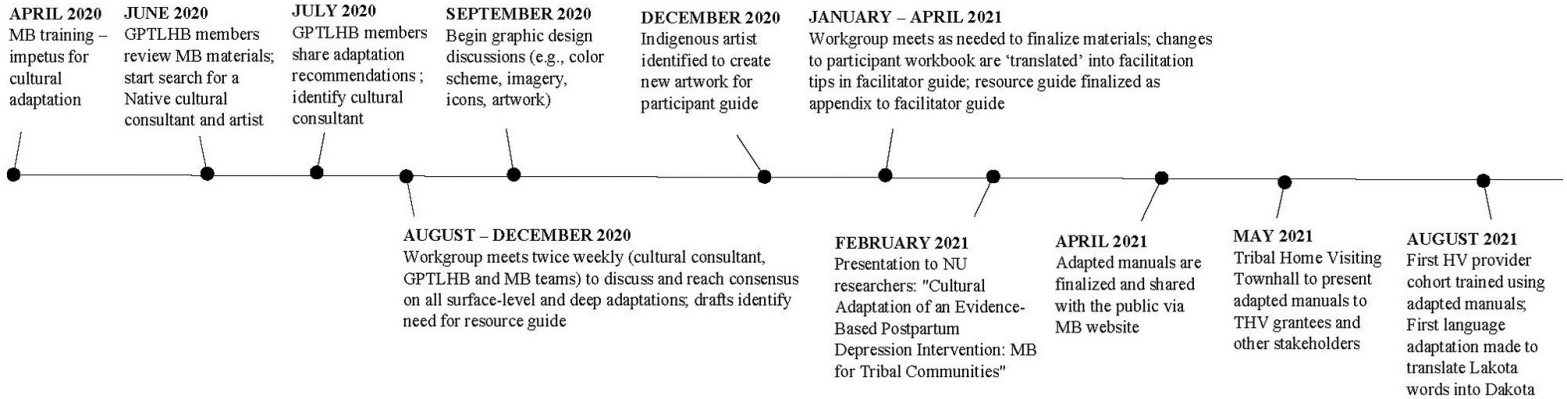
Timeline of cultural adaptation workgroup process and dissemination to date.

Program managers and staff from GPTLHB reviewed the MB participant and facilitator manuals to identify areas where cultural adaptation was needed, including artwork, images, design elements, and cultural and spiritually grounded content. Family-facing materials were prioritized to ground the adaptations within the participant experience. The next phase of adaptations was guided by the cultural consultant, who shared traditional teachings with the group as we learned and discussed how to integrate core concepts and practices to promote connectedness with Lakota culture (see Figure 1 for timeline). Throughout the collaborative process we discussed ways to incorporate cultural components into the cognitive, behavioral, and attachment foundations of the MB intervention.

Adaptations were focused on representation of the Lakota people, who are part of the Oceti Sakowin (Seven Council Fires). The workgroup allowed space and time to process the intersection of scientific knowledge with Indigenous knowledge in determining how best to integrate the two for optimal fit and impact, navigating through a “cultural interface” (52). The workgroup attended to the challenges of creating cultural congruence by reflecting the core values we promote in the intervention. Our process reflected *humility* among those who were learning about Lakota beliefs and traditions, *respect and honor* for elders and service providers who shared traditional knowledge and experiences, *wisdom* guiding preservation of the core MB intervention to maintain its fidelity as well as relatability for both providers and participants, *compassion* for American Indian children, families and communities, *generosity* of time, effort, and expertise, and openness throughout the iterative process to reach consensus on every adaptation.

### Cultural Context Informing Adaptation

Traditionally and historically, Lakota people believe in the sacredness of life. A prime example of this was how Lakota children were viewed—as gifts from the Creator and as the life blood of the future of the people. Thus, every opportunity to enhance their growth—physically, mentally, emotionally, and spiritually—was taken as a responsibility to support them in reaching their full potential and fulfill their purpose in life. Lakota people refer to the unborn child as “*hoksi nagi*” (spirit infant) because it is believed that the child exists as a spirit being prior to being born to the earth. The mother was protected by the extended family thereby protecting the hoksi nagi. Once the hoksi nagi came to the physical realm of the earth, then they traveled through four stages of life called “Oinajin Topa” and within those four stages there were seven transitions that each individual made.

Each of the seven transitions had teachings, protocols, and ceremonies that corresponded to the age range within each stage. For example, the *Hoksicila* (infant) stage began with a grandmother welcoming the infant to the world, bestowing a blessing on the infant, giving thanks to the Creator for the gift, and making a prediction for the life of the infant as they make the journey through each stage. The *Wakanyeja* (as a sacred being or child) was treated as sacred, never being hit and always spoken to gently, which was a reflection of the belief of their sacredness. Conduct toward the child was governed by the belief that the child's spirit could turn around and return to the spirit world at any time. The epistemology, or ways of knowing, of the Lakota people included great reverence for “allegiance to higher spiritual powers” (53). The belief that those higher spiritual powers could influence the journey of the child on earth or contribute to an untimely return to the spirit world was given great credence. Unfortunately, the disconnection of many Lakota people from their cultural and spiritual lifeways has resulted in some children no longer being treated as sacred beings. The disproportionately high rates of abuse and family separation among American Indian children illustrate the glaring departure from the concept of children as gifts from the Creator to be treated as sacred and shows the need to reconnect to that concept (10).

How do we reconnect to the conceptual understanding that children are sacred and the relationship between the mother and her baby requires ongoing support? The cultural adaptation of MB aimed to underscore the need for that reconnection. While it was the Lakota worldview, belief system and ancestral teachings that informed the cultural adaptation, it was understood there is a great diversity among American Indians so key constructs that spoke to universally held beliefs were highlighted; first and foremost, “children are sacred” has been a common belief among many, if not all, American Indian tribal nations. Warne (54) posits that strengthening cultural values is an important strategy to reducing health disparities, while highlighting the critical factor of the impact that family health has on child health. The cultural adaptation of MB weaves activities throughout that strengthen the bond between the mother and her culture, thereby strengthening the bond between the mother and her baby. The cultural adaptation of MB was a deliberate and thoughtful effort toward cultural grounding of the material, always with the sacredness of children in the forefront and Lakota cultural ancestral teachings as the foundation.

## Results

### Description of Adaptations

After thorough review of the participant and facilitator manuals by the entire workgroup, various changes were made to both manuals, inclusive of surface- and deep-level adaptations. Adaptations began with the participant manual given that these materials are family-facing. Adaptations were then made to the facilitator guide to reflect changes in the participant guide, and to provide further resources and support for service providers when delivering the adapted curriculum (see Table 2 for description).

**Table 2:** Adaptations.

**Manual**	**Section**	**Type of adaptation**	**Adaptation description**
Both	Throughout	Surface-level:	**Color scheme** changed throughout entire participant and facilitator manual, beading motif added throughout
			**Language—Lakota words** included *throughout* both manuals (i.e., Ina Na Hoksicila Woonspe = The Mothers and Babies Course) (those that have deep spirit and cultural roots have been included below, all language changes have not been listed due to sheer amount)
			**Culturally appropriate illustrations:** imagery changed throughout manuals to be representative of native peoples (individual changes of this type are not all listed below due to sheer amount)
Both	Cover	Surface-level and Deep:	**Culturally appropriate illustration—cover art** changed to artwork of a Lakota star quilt on both manuals
Participant	Introduction, Session 1	Surface-level:	**1.1: tailoring content**—“too much work” changed to “employment issues; and “household chores” changed to, “lack of safe, adequate, and stable housing,” “problems with your partner or others” changed to ‘relationship issues”
			**1.4: culturally appropriate illustration**—seesaw changed to tipping canoe
			**1.5: tailoring content**—slight modification to streamline “what is mindfulness?” content
		Deep:	**1.2: new worksheet added**—“Strengths that can affect the mother–baby relationship”
			**1.3: new worksheet added**—“Woope Sakowin—Seven Sacred Laws”
			**1.5: tailoring content**—cultural references added linking mindfulness to seven sacred laws (i.e., “inila”-being still, calm, quiet; “ksapa” being aware/alert)
			**1.6: tailoring content**—Lakota worldview and medicine wheel included to incorporate spirit into inner reality
Participant	Pleasant Activities, Session 2	Surface-level:	**2.1: culturally appropriate illustration, tailoring content—**Dawn and Sunset vignette, pleasant activity = making wojapi
			**2.2: culturally appropriate illustration, tailoring content—**pleasant activities list changed to include culturally appropriate activities, illustration of native family added
			**2.3: illustration**—illustration changed to tree local to Lakota region
		Deep:	**2.3: culturally appropriate illustration**—tree has been adorned with prayer tobacco tie offerings (used during Sun Dance)
Participant	Pleasant Activities, Session 3	Surface-level:	**3.1: culturally appropriate illustration, tailoring content—**“obstacles” changed to “challenges,” illustration of woman climbing stairs changed to person at fork in road in nature
Participant	Thoughts, Session 4	Deep:	**4.1: tailoring content—**Thought bubble changed from “Pregnancy and having a new baby are special times in my life...” to “A new baby is a blessing from the Creator”
			**4.3: tailoring content—**instructions changed to “Use the mind's eye”
		Surface-level:	**4.2: culturally appropriate illustration**—Dawn and Sunset vignette
			**4.3: photograph—**photograph of maple leaves (local to Lakota region)
Participant	Thoughts, Session 5	Surface level:	**5.3: illustration**—illustration changed to tree local to Lakota region
		Deep:	**5.3: culturally appropriate illustration**—tree has been adorned with prayer ties (used during Sun Dance)
Participant	Thoughts, Session 6	Deep:	**6.2: tailoring content and imagery—**Thinking about baby's future reimagined as concentric circles and a progression over the first 5 years of child's life
Participant	Session 6	Surface-level:	**6.3: culturally appropriate illustration and tailoring content:** Example and corresponding illustrations changed to “I want to have my own home” and “I will connect with housing resources in my community”
Participant	Contact With Others, Session 7	Surface-level:	**7.2: culturally appropriate illustration and tailoring content—**Dawn and sunset vignette, activity changed from shopping to taking a walk
Participant	Session 7	Deep:	**7.3: tailoring content—**spirit and cultural elements incorporated into mindfulness practice (i.e., “Reach up to ANPE WI (Sun). Envision the WICAHPI OYATE (the Star Nation) and UNCI MAKA (Grandmother Earth). Remember you are a part of the MAKA SITOMNI (Universe) and were sent from the NAGI YATA (Spirit World) as a blessing to the people and this earth”)
Participant	Contact With Others, Session 8	Deep:	**8.1: tailoring content and imagery—**“The People In My Life” graphic reimagined as a circle of kinship incorporating visual elements from the seven sacred laws
Facilitator	Throughout		**Instructions** changed to incorporate Seven Sacred Laws, Sacred Circle Within Me and all other new additions
			**Medicine wheel icon** used throughout to demonstrate when an adaptation was made and/or where a facilitator can make further adaptations to meet the needs of their community
Facilitator	Appendices		**Appendices** added at end of facilitator manual including: (a) Cultural Adaptation Process and Workgroup Members, (b) Glossary, (c) Seven Sacred Laws (extra worksheet for printing), (d) Sacred Circle Within Me (extra worksheet for printing), (e) How To Make A Wokpan (Spirit Toolkit), (f) Kinship Chart, (g) Oglala Lakota Spirit Calendar, (h) Adaptation Guidance, and (i) Additional Resources

Surface-level adaptations consisted of revised color scheme, cover artwork depicting a star quilt, photos, and illustrations depicting Indigenous peoples, clothing, activities, and culture, and Lakota language for key concepts (e.g., sacred laws, kinship terms). For example, in the original MB participant manual, a seesaw depicts the imbalance that stress causes in our lives, vs. the balance that is created when we utilize the cognitive-behavioral skills learned in MB. This seesaw was replaced with a tipping and then balanced canoe. Three vignettes introduce each core CBT module through the stories of Violet and Mary's days. These were reimagined as Dawn and Sunset—names reflecting the connection with the natural world common among Lakota people. We worked with an artist of Colombian and Honduran native descent, to create appropriate illustrations. Pleasant activity examples were changed to be more culturally appropriate, such as attending a pow wow, harvesting herbs, and beading. Each of these elements were selected after deliberation and consensus among workgroup members, using an iterative process.

Deep-level adaptations incorporated important spiritual elements, ancestral teachings, and cultural values. In the first MB session, as participants are introduced to stressors that can affect the mother–baby relationship, they are also introduced to the Lakota worldview of governance of self, family, and community referred to as Woop'e Sakowin (Seven Sacred Laws)—compassion, generosity, humility, fortitude, respect and honor, bravery, and wisdom. An additional worksheet addressing strengths that can affect the mother–baby relationship encourages participants to identify strengths they already have. Examples such as “wocekiye” (sending voice to Creator) and “wotakuye” (strong kinship system) are included to support the process of identifying with culturally-based strengths and values. As participants are introduced to their personal reality—a term used to encompass both inner and outer experiences in relation to cognitive-behavioral theory—a lens toward the Lakota worldview “woiwanke” is used to frame concepts. A medicine wheel or sacred circle is used to depict the relationship between our inner and outer realities, and our mood. The Lakota worldview includes “nagi” or spirit within one's inner reality, encouraging participants to strengthen the core part of their being—their spirit. Each of these elements are revisited throughout the curriculum to reinforce that spirituality and the seven sacred laws are fundamental elements of reducing stress, improving mental health, and building a healthy mother–baby relationship.

A small medicine wheel icon is used throughout the facilitator manual to indicate where adaptations have been made and places that further adaptations can be made to meet the needs of a particular community. Several appendices were added to the facilitator guide with further cultural teachings and practices such as “How to Make a Wokpan” (spirit toolkit), a kinship chart, and a spiritual calendar.

### Description of Products

Fully adapted participant and facilitator MB manuals for use in tribal communities are available for access *via* the MB website^1^. Adaptation guidance is provided through facilitation tips within MB sessions referencing cultural resources and approaches, as well as an adaptation guide in the appendix to provide context and guidance for making further fidelity-consistent cultural adaptations. The adaptation guide includes: (1) “green light” adaptations—safe, encouraged, and fidelity consistent, to fit the culture and context; (2) “yellow light” adaptations—changes that should be made with caution, consulting an expert is encouraged; and (3) “red light” adaptations—fidelity inconsistent changes that would weaken or remove key components of the intervention and would therefore compromise intervention fidelity and efficacy. While this adaptation was developed with and for members of the Lakota Tribe, we believe that these culturally adapted MB manuals will be useful in other tribal communities as well, with further adaptation encouraged to ensure cultural fit, acceptability, and sustainable implementation.

### Dissemination

Since the completing this cultural adaptation, we have co-facilitated a presentation with the US Health Resources and Services Administration, to which Tribal Maternal, Infant and Early Childhood Home Visiting Program grantees were invited, sharing background on MB, the cultural adaptation process, and examples of adapted content. We then facilitated a MB training for a group of service providers from organizations serving Indigenous populations in North Dakota, South Dakota and Michigan. A home visiting program in South Dakota translated the Lakota language into Dakota, to fit their community. Maternal and child health leadership in Vermont are discussing next steps in adapting MB for the Abenaki families they serve, and other home visiting networks serving American Indian families and communities have expressed interest in being trained in the culturally adapted manuals. This early interest and uptake is a testament to the purpose and the process for which our workgroup advocated.

## Discussion

If successful, adapted EBIs can have profound impact by improving reach, effectiveness, and health equity for marginalized populations. This cultural adaptation of MB has the potential for significant impact and is well-positioned to improve access to mental health resources as it can be implemented by paraprofessionals (39, 41). Moving tasks, as appropriate, to staff with lesser training, (“task-shifting”) (55), can empower Indigenous paraprofessionals who are familiar with the spirituality, kinship patterns, language, and historical collective trauma to provide their participants with tools and skills to address their mental health. This adapted version of MB has the potential to reduce depressive symptoms, improve mother–baby attachment, reinvigorate engagement with spirit and cultural practices, and promote overall well-being of Indigenous families.

This adaptation process, grounded in humility and compassion, may be replicated by future scholars in collaboration with community members as they wish to adapt EBIs for varying populations and contexts. To prepare for these adaptations, researchers and intervention developers can proactively identify thematic elements in an EBI that must remain in order to maintain fidelity. Cultural adaptations must be led by members of the community. We encourage scholars and researchers engaging in this process to do so with intention, openness, compassion, and a focus on health equity.

### Evaluation

This work is intended to enhance the acceptability, reach, and uptake of the MB intervention, to improve mental health outcomes of parents and children through an adapted EBI that prioritizes cultural restoration alongside cognitive-behavioral skills. The use of hybrid effectiveness-implementation research designs (56) can guide aspects of both intervention implementation and effectiveness evaluation. We will evaluate the acceptability, feasibility, and fidelity of the adapted manuals through yearly implementation surveys, developed to guide scaling among tribal communities and through tribal home visiting. Additional implementation metrics include qualitative research with service providers and participants, to learn more about intervention and adaptation acceptability, skill utilization, participant responsiveness, and provider recommendations. We will collaborate with Indigenous communities to develop culturally relevant measures and acceptable methods to evaluate the implementation and impact of the culturally adapted intervention, to promote acceptability and effectiveness of the research process as well (57).

### Future Directions

There is need for further funding for development and evaluation of culturally-normed EBIs in Tribal communities, further cultural adaptation of EBIs, as well as development of EBIs specifically for and with Indigenous populations across healthcare and service systems.

## Data Availability Statement

The original contributions presented in the study are included in the article/supplementary material, further inquiries can be directed to the corresponding author/s.

## Author Contributions

All authors listed have made a substantial, direct, and intellectual contribution to the work and approved it for publication.

## Funding

We would like to thank the following funders for their support for this cultural adaptation: Substance Abuse and Mental Health Services Administration Grant #1 H79 SM080173-01 to the Great Plains Tribal Leaders' Health Board; Perigee Fund grant to the Northwestern University Mothers and Babies Program (PI: SD Tandon).

## Conflict of Interest

The authors declare that the research was conducted in the absence of any commercial or financial relationships that could be construed as a potential conflict of interest.

## Publisher's Note

All claims expressed in this article are solely those of the authors and do not necessarily represent those of their affiliated organizations, or those of the publisher, the editors and the reviewers. Any product that may be evaluated in this article, or claim that may be made by its manufacturer, is not guaranteed or endorsed by the publisher.

## Footnotes

^1^ mothersandbabiesprogram.org
